# Plasma-activated gas: a new dawn for wound care and infection

**DOI:** 10.4103/mgr.MEDGASRES-D-25-00282

**Published:** 2026-04-11

**Authors:** Xiang Li, Ya-Shi Zhong, Ming-Yang Yuan, Zhen-Ling Xie, Xin-Rui Zhang, Zi-Zhu Zhang, Ji-Shen Zhang, Dong-Ming Ren, Ding-Xin Liu, Ming-Xiao Hou, Yun-En Liu

**Affiliations:** 1 School of Shuren International (School of Innovation and Entrepreneurship), Shenyang Medical College, Shenyang, Liaoning Province, China; 2 Liaoning Provincial Key Laboratory of Skin, Mucosa and Soft Tissue Trauma Repair and Reconstruction, Shenyang, Liaoning Province, China; 3 Shenyang Municipal Key Laboratory of Digestive System Injury Repair and Reconstruction, Shenyang, Liaoning Province, China; 4 School of Graduate, Shenyang Medical College, Shenyang, Liaoning Province, China; 5 Center for Plasma Biomedicine, Xi’an Jiaotong University, Xi’an, Shaanxi Province, China; 6 Department of Intervention, Ansteel General Hospital, Anshan, Liaoning Province, China

**Keywords:** angiogenesis, anti-infection, antimicrobial activity, chronic wound, cold plasma technology, plasma-activated gas, reactive oxygen and nitrogen species, signaling pathways, tissue remodeling, wound healing

## Abstract

**Facts**

1. Physical plasma generates reactive oxygen and nitrogen species (RONS), exerting antitumor, anti-infective, and tissue-modulating effects in models.
2. Plasma-derived reactive species act on cell membranes to trigger sustained biological signals.
3. Computational modelling combined with experimental clarify of RONS transport, membrane interactions, and downstream signaling in diseases.
4. Plasma RONS work alone or sensitize other therapies via oxidative stress and tissue remodeling.
5. Plasma modulates immunity: redox signaling, danger-associated molecular pattern release, and immune activation drive durable antitumor responses.

**Open Questions**

1. Which RONS drives beneficial/detrimental effects, and how to precisely control them clinically?
2. How do membrane lipid features affect plasma-cell interactions? Do tumor membranes explain selective plasma sensitivity?
3. What core signaling networks follow plasma-induced membrane oxidation, and how do pathways (mitogen activated protein kinases, nuclear factor-κB, etc.) interact in diseases?
4. Can standardized biomarkers predict response/safety when plasma is combined with other therapies?
5. How to integrate computational modeling with experiments/clinical data for personalized plasma medicine?

Plasma-activated gas, an emerging technology derived from non-thermal plasma, demonstrates considerable promise in wound management through its rich repertoire of reactive oxygen and nitrogen species. Plasma-activated gas exerts broad-spectrum antimicrobial activity through multi-target mechanisms, effectively eliminating multidrug-resistant pathogens, including methicillin-resistant *Staphylococcus aureus* and *Pseudomonas aeruginosa*, as well as disrupting bacterial biofilm architecture. In tissue repair, plasma-activated gas orchestrates the entire wound healing cascade. It accelerates platelet activation and coagulation, enhances proliferation and migration of epithelial cells and fibroblasts by activating vascular endothelial growth factor receptor-extracellular regulated protein kinases 1/2 and transforming growth factor-β/Smad signaling pathways, promotes angiogenesis via the endothelial nitric oxide synthase–vascular endothelial growth factor axis, and optimizes tissue remodeling by fine-tuning the matrix metalloproteinases and their tissue inhibitors balance. Preclinical and early clinical studies confirm that plasma-activated gas significantly shortens healing time, mitigates scar formation, and effectively controls wound infection. Although challenges remain in standardization, safety profiling, and clinical translation, advances in precise dosing strategies and intelligent device design position plasma-activated gas as a transformative approach for managing refractory chronic wounds. The results and cutting-edge advancements summarized in this review are expected to provide a valuable and forward-looking reference for the subsequent research and development upgrading and clinical translational application of plasma-activated gas.

## Introduction

### Current landscape and challenges in wound healing and anti-infection therapy

Wound healing is a precisely orchestrated biological process involving hemostasis, inflammation, proliferation, and remodeling. However, managing chronic wounds remains complex and challenging.^1^ Infection and related complications are central factors impeding healing in chronic wounds. Local or systemic infections, often involving multidrug-resistant bacteria, substantially compromise the efficacy of conventional antimicrobial therapies. Systemic antibiotics frequently fail to penetrate biofilms effectively, while topical antibiotics are limited by insufficient efficacy, potential to induce resistant strains, and risks of allergic or sensitization reactions. Consequently, antibiotics and antiseptics combined with wound dressings are increasingly regarded as unsuitable for routine topical use.^2^

Moreover, existing wound dressings exhibit notable limitations. Conventional gauze adheres to the wound bed upon exudate absorption, causing secondary trauma and pain upon removal.^3^ Advanced dressings, such as hydrogels, hydrocolloids, and hydrofibers, maintain a moist environment, alleviate pain, and facilitate drug permeation; yet, in severe burns, eschar formation impedes passive diffusion of therapeutic agents, markedly reducing their effectiveness.^4^ Critically, although a moist environment supports tissue repair, it can also promote microbial proliferation, increasing the risk of secondary infection. Once established, infections exacerbate patient morbidity, prolong hospitalization and recovery, and substantially elevate treatment costs.^5^ In comparison, plasma-activated gas (PAG) fills these gaps and has obvious advantages over standard dressings: (1) Treatment depth: The gas-active substances provided by PAG can diffuse to the infection depth through the dense wound structure, overcoming the penetration limitations of liquid dressings. (2) Speed of effect: Traditional and advanced dressings rely on the gradual release of drugs to achieve therapeutic effects, while PAG directly exerts rapid antibacterial and anti-inflammatory effects, avoiding the delayed onset of effect of standard dressings. (3) Easy operation: PAG adopts a non-contact application method, eliminating the risk of trauma related to gauze adhesion. It also does not require a dedicated storage for the dressing itself, simplifying clinical use compared to bulky or stable-sensitive advanced dressing products.

In recent years, plasma-based technologies have gained attention in wound management. Plasma-activated water, for instance, has demonstrated pro-healing effects in animal studies and exhibits favorable safety profiles as a skin disinfectant. However, plasma-activated water stability is highly pH-dependent: under near-neutral conditions, reactive species such as hydrogen peroxide (H_2_O_2_), nitrite anion (NO_2_^−^), nitrate anion (NO_3_^−^), and peroxynitrite anion (ONOO^−^), remain stable, whereas under strongly acidic conditions (pH < 3.5), these species become unstable due to mutual reactions.^6^ In addition, plasma-activated water relies on liquid contact delivery and has difficulty penetrating wound exudates and exerting deep tissue functions, which limits its therapeutic effect in complex infected wounds.

In contrast, PAG has emerged as a highly promising alternative. Generated via low-temperature plasma technology, PAG is enriched with reactive oxygen and nitrogen species (RONS), including high-valent nitrogen oxides (NO_x_, such as dinitrogen pentoxide) and short-lived free radicals, such as ONOO^−^ and superoxide anion (O_2_^−^). Compared with plasma-activated water, they exhibit superior stability in the physiological environment and can stably accumulate in wound exudates. PAG has broad-spectrum antibacterial, anti-inflammatory, and tissue regeneration functions: *In vitro* experiments have confirmed that PAG can inactivate *Staphylococcus aureus* (*S. aureus*) within 6 minutes. *In vivo* studies have shown that a 3-minute daily treatment can reduce the bacterial load on the wound by ≥ 90% on the second day and simultaneously inhibit macrophage infiltration.^7^ Induce the production of fibroblast growth factor, epidermal growth factor, and vascular endothelial growth factor (VEGF), and accelerate healing.^8^ Compared with conventional antibiotic therapies, PAG offers distinct advantages: its multi-modal antimicrobial action, including bacterial membrane disruption, biofilm inhibition, and induction of bacterial apoptosis, considerably reduces the risk of resistance development. PAG also modulates the local wound microenvironment by attenuating inflammatory cytokine release and promoting angiogenesis, thereby accelerating healing. Furthermore, its non-contact nature and controllable application enhance clinical safety, particularly in complex and chronically infected wounds.^7^,^8^

Evidence indicates that these bioactive molecules, generated by gas plasma under near-physiological temperatures, can regulate the migration of immune cells, such as neutrophils, macrophages, and lymphocytes, enhance antimicrobial immunity, and coordinate inflammation resolution with tissue regeneration, offering a novel therapeutic opportunity for refractory wound healing.^9^

### Conception and research significance of plasma-activated gas

Gas plasma, a partially ionized gas or vapor regarded as the fourth state of matter, is ubiquitous throughout the universe, occurring in stellar and nebular phenomena, and manifesting in natural events such as lightning, St. Elmo’s fire, and corona discharges. Under atmospheric pressure conditions, gas plasmas are categorized into thermal and non-thermal (cold) types, formed when electrons and ions are rapidly heated under strong electric fields or energy excitation, leading to gas ionization.^10^ The properties of plasma are governed by key parameters, including electron density and electron energy distribution, with electron temperature serving as a critical determinant. Variations in gas composition significantly influence the profile and relative abundance of reactive species in the plasma, thereby diversifying its physicochemical and biological effects. Typically, gas plasmas maintain a state of quasi-neutrality and stability due to a relative balance between ions and electrons, a fundamental precondition for their biological efficacy.^11^

PAG exhibits multiple medical applications. Plasma-activated solution, generated through cold plasma treatment, retains a variety of reactive species, such as radicals, metastable atoms, and oxygen-, nitrogen-, or carbon-containing reactive groups, formed via electron–ion interactions, and demonstrates considerable bioactivity, positioning it as a novel therapeutic modality.^12^ The plasma jet pen, utilizing cold plasma, produces reactive chemical species with selective activity against cancer cells, enabling direct application to exposed lesions during surgery or external treatment. In surgical contexts, thermal plasma achieves hemostasis and vessel sealing through coagulation, minimizing thermal damage and underscoring its utility in operative settings.^11^

RONS-based therapies have attracted increasing attention for their targeted action in chronic wound management. Studies indicate that RONS, delivered via gas plasma technology under near-physiological temperatures, enter the wound microenvironment and support sequential recruitment of neutrophils, macrophages, and lymphocytes. This facilitates infection clearance, enhances antimicrobial immunity, and coordinates inflammatory resolution with tissue regeneration.^9^ Plasma treatment has been shown to significantly improve oxygenation in both superficial and deep wound tissues, elevate hemoglobin and tissue water indices, and promote microcirculation and tissue repair.^13^ In infection control, cold atmospheric argon (Ar) plasma has been established as a safe, effective, and painless adjunct therapy for drive-line infections, capable of reducing pathogen load without increasing reoperation risk or causing significant peri-tissue damage.^14^ The cold atmospheric plasma jet, a non-thermal plasma variant, has been widely investigated for its dual antibacterial and pro-healing functions. Helium (He)/Ar-based cold atmospheric plasma jet promotes keratinocyte proliferation and migration by activating epithelial–mesenchymal transition and cell cycle pathways, evidenced by reduced expression levels of E-cadherin and elevated N-cadherin, CyclinD1, Ki-67 antigen, cyclin-dependent kinase 2, and phosphorylated extracellular regulated protein kinase. Animal studies confirm that He/Ar-cold atmospheric plasma jet enhances granulation tissue formation, attenuates local inflammation, and significantly accelerates wound closure.^15^

In oncology, gas plasma has demonstrated considerable research value. Initial evidence of its antitumor effects emerged in animal studies in the early 2010s. Medical gas plasma facilitates the deposition of diverse physical and chemical active mediators, particularly RONS, on tissue surfaces, enabling direct tumor cell killing and microenvironment modulation.^16^ Recent work further reveals that plasma-derived therapeutic RONS exert multifaceted antitumor effects, both as monotherapy and in combination with radiotherapy, chemotherapy, or immunotherapy.^17^,^18^ Notably, plasma technology can induce immunogenic cell death, conferring immune-stimulatory effects that may enhance current cancer immunotherapies via *in situ* or autologous tumor vaccination strategies.^16^ In breast cancer models, plasma therapy not only directly suppresses tumor growth but also augments anti-tumor immune responses, suggesting novel avenues for therapeutic development.^19^ Atmospheric pressure plasma is increasingly recognized as a valuable adjunct to conventional cancer therapies, improving selectivity and efficacy against resistant tumors when combined with radio-, chemo-, or immunotherapy.^18^

A growing body of evidence characterizes plasma-derived reactive species, from their spatiotemporal distribution in the gas phase to the complex chemistry and biochemical responses following dissolution into liquids. Computational modelling and data analysis have identified potential interaction pathways between plasma-generated reactants and biological membranes, with experimental validation across cellular and animal disease models gradually elucidating the molecular mechanisms of physical plasma. In this process, the cell membrane has been recognized as a critical interface where plasma-generated reactive oxygen species (ROS) transition from chemical reactivity to diverse biological effects.^20^,^21^ A milestone was reached in 2013, when the first medical plasma systems entered the European market as Class IIa medical devices, marking a pivotal step in the clinical translation of plasma medicine. These systems have proven to be valuable resources in dermatology, particularly in supporting the healing of chronic wounds.^19^

Beyond wound repair and oncology, PAG exhibits promise in other medical domains. For instance, novel argon PAG has demonstrated remarkable efficacy in disinfecting endoscope channels and potential sterilization applications. Experimental results show that a nine-minute PAG treatment markedly reduces viable bacterial counts in biofilms formed on polytetrafluoroethylene channels, disrupts biofilm integrity, inhibits its regrowth, and preserves material morphology, highlighting its potential for reprocessing medical devices.^22^

As an emerging and rapidly evolving discipline, plasma medicine is progressively transitioning from basic research to clinical practice. Currently, plasma-based devices are employed in surgical instrument sterilization and the management of chronic and infected wounds, with applications spanning wound healing, disinfection, oncology, and dentistry.^21^,^23^,^24^ With ongoing research and technological refinement, the medical applications of PAG are poised for continued expansion.

This review summarizes principles, generation tech/equipment, active substances, healing/anti-infection effects, mechanisms, challenges, and prospects of PAG in wound management. It aims to provide a systematic and in-depth reference for subsequent research on PAG, promote the upgrading of their development technology, and accelerate their clinical conversion application in the management of refractory chronic wounds.

## Retrieval Strategy

To achieve this objective, a comprehensive literature search was conducted via PubMed and ScienceDirect. The following terms were used: (“plasma-activated gas” OR “cold atmospheric plasma gas” OR “non-thermal plasma gas”) AND (“wound healing” OR “chronic wound” OR “refractory wound” OR “antimicrobial activity” OR “multidrug-resistant pathogen” OR “bacterial biofilm” OR “tissue repair” OR “angiogenesis” OR “plasma generation technology” OR “reactive oxygen and nitrogen species” OR “clinical translation”). The inclusion criteria were: (1) Original research articles, systematic reviews, meta-analyses, and preclinical/clinical trial reports that directly address the biological effects, mechanisms, technical development, or therapeutic applications of PAG in wound management. (2) Studies published in English between the years 1994 and 2025. (3) Publications with complete experimental design, clear data presentation, and relevant outcome indicators. The exclusion criteria were: (1) Articles not available in full text. (2) Conference abstracts, case reports, editorials, or review articles lacking original data support.

## Sources of Plasma-Activated Gas

### Fundamental principles of gas-to-plasma transition

The transition from gaseous to plasma state fundamentally represents a non-equilibrium process in which a gas undergoes partial ionization under external energy input, such as electric fields, radiofrequency/microwave electromagnetic fields, or thermal sources. During this process, accelerated electrons engage in inelastic collisions with neutral molecules or atoms, initiating mechanisms including electron-impact ionization, Townsend avalanches, stepwise ionization, and Penning ionization. These events collectively drive a rapid increase in free electron and ion densities. A self-sustained discharge, and thus the plasma state, is achieved when the electron multiplication rate from primary and secondary electron emission surpasses the loss rate, satisfying the breakdown criterion. In low-temperature plasmas, the electron temperature typically far exceeds the gas temperature, while the system maintains overall quasi-neutrality and is governed by Debye shielding and electromagnetic confinement. These characteristics critically determine subsequent energy coupling, reaction kinetics, and species transport.^25^,^26^,^27^

At atmospheric pressure, stable non-thermal plasma discharges with uniform plasma zones can be achieved using inert gases (e.g., He/Ar) or gas mixtures, alongside pulsed/radio frequency excitation and pre-ionization strategies. These approaches establish the discharge physics foundation for generating PAG and atmospheric pressure plasma jets.^25^,^28^,^29^,^30^ From an application perspective, the coupling between gas-phase discharges and interfaces or liquid phases directly governs the flux, lifetime, and selectivity of reactive species, e.g., atomic oxygen (O), hydroxyl radical (OH), ozone (O_3_), and NO_x_, thereby determining the efficacy and reproducibility of PAGs in sterilization, surface modification, and biomedical applications.

### Common plasma generation technologies

#### Direct current glow discharge

A constant high voltage is applied between anode and cathode in low-pressure gas, where electrons gain energy in the cathode sheath and sustain ionization in the positive column. This simple configuration facilitates the study of elementary processes and plasma–surface interactions, and is widely used in compact plasma sources and pretreatment units. Under atmospheric pressure or small-gap conditions, pre-ionization or pulsed strategies enhance discharge uniformity and ignition characteristics.^30^

#### Radio frequency capacitively coupled plasma

Operating typically at 13.56 MHz, energy is coupled into the plasma via parallel plate electrodes. Dual-frequency excitation (e.g., 13.56/2 MHz) enables independent control over plasma density and ion energy, improving process uniformity and the operational window. Commonly employed in low-pressure plasma cleaning, etching/ashing, and surface activation.^31^

#### Inductively coupled plasma

An external induction coil generates an induced electric field in a vacuum chamber, primarily heating electrons inductively. Inductively coupled plasma systems offer high plasma density with low ion bombardment energy. Combined with an independent bias source, they allow decoupled control of plasma density and ion energy, making them suitable for high-anisotropy etching and high-throughput surface processing.^32^

#### Microwave/electron cyclotron resonance plasma

Using microwave frequencies such as 2.45 GHz, these sources achieve efficient electron energy absorption via electron cyclotron resonance under matched magnetic field conditions. They enable high-density, low-pressure plasma generation and are applied in plasma cleaning, activation, and selected fine deposition/etching processes.^33^

#### Dielectric barrier discharge

By inserting dielectric layers between at least one electrode and the discharge gap, arc formation is suppressed, enabling non-equilibrium discharge operation. Dielectric barrier discharge operates stably at atmospheric pressure with diverse configurations (volume- or surface-type), serving as a leading technology for atmospheric plasma activation and continuous surface treatment.^34^

#### Atmospheric pressure plasma jet

Using coaxial, needle-ring, or dielectric-coated nozzles, these devices generate a plasma plume in ambient air, delivering both short- and long-lived reactive species to target surfaces. Nozzle geometry, working gas composition, and excitation waveforms influence ionization wave propagation, chemical field distribution, and species flux. Atmospheric pressure plasma jets are widely used in surface modification, contaminant removal, and activated gas generation.^25^,^35^

#### Corona discharge and other atmospheric sources

These rely strongly on inhomogeneous electric fields (e.g., needle–plane electrodes) to achieve localized discharge. Simple in structure and fast in response, they are commonly used in denitrification/purification and online surface treatment, and represent one route to generating activated gases.^35^

### Typical equipment for plasma cleaning and surface treatment

Vacuum plasma cleaners typically employ capacitively coupled plasma, inductively coupled plasma, or electron cyclotron resonance sources, introducing process gases such as oxygen or argon into evacuated chambers. Through combined physical sputtering and chemical reactions, they remove organic contaminants and introduce functional groups, enabling uniform and reproducible processing of morphology-sensitive and heat-sensitive materials.^33^ Atmospheric plasma cleaning/activation systems often adopt dielectric barrier discharge or atmospheric pressure plasma jets configurations, providing vacuum-free, online surface activation and decontamination. These are particularly suited for roll-to-roll processing and large-area, irregularly shaped components, and can function directly as PAG generation and delivery units.

The summary of gas-to-plasma conversion principles and common generation equipment & technologies can be seen in **Table 1**.^25^,^26^,^27^,^28^,^30^,^31^,^32^,^33^,^34^,^35^,^36^

**Table 1:** A summary of gas-to-plasma conversion principles and common generation equipment & technologies

Category	Specific type	Core principle/technical feature	Key parameter/influencer	Application	Reference
Gas-to-plasma conversion principles	Conversion essence and self-sustained discharge	1. External energy (electric field, RF/microwave electromagnetic field, heat source) partially ionizes gas; inelastic collisions between electrons and neutral particles trigger ionization (electron impact ionization, Townsend avalanche, stepwise ionization, Penning ionization). 2. When electron multiplication rate > loss rate, breakdown condition is met, discharge becomes self-sustained, and gas turns into plasma. 3. Low-temperature plasma characteristics: electron temperature (*T*_e_) much higher than gas temperature (*T*_g_), quasi-neutrality, subject to Debye shielding and electromagnetic confinement.	1. Type and intensity of external energy. 2. Electron collision frequency and ionization efficiency. 3. Gas type (inert gases maintain stable discharge easily).	Provides physical basis for various plasma generation equipment; determines subsequent generation and reaction kinetics of active species.	25
	Breakdown characteristics and discharge stage evolution	1. Breakdown voltage is described by Paschen’s law and its modified forms, related to pressure-gap product (*pd*), with a minimum breakdown voltage. 2. Under extreme conditions (micro/nano gaps, high pressure), field emission and surface effects deviate from classical Paschen curve. 3. Macroscopic discharge stages: Townsend discharge → glow discharge → arc discharge; electric field distribution, sheath structure, and energy deposition mode differ significantly in each stage.	1. Gas pressure (*p*) and electrode gap (*d*). 2. Gap size and pressure. 3. Applied voltage and current density.	Guides electrode design and gas pressure control of plasma equipment; avoids discharge transitioning to arc in non-thermal plasma applications.	26 27 28
	Atmospheric pressure non-thermal plasma stabilization technology	Stable and uniform non-thermal plasma discharge is achieved by using inert gases (He/Ar) or mixed gases, pulse/RF driving, and pre-ionization technology to suppress arc formation.	1. Working gas type (inert gases like He/Ar). 2. Driving waveform (pulse/RF). 3. Pre-ionization intensity.	Provides stable discharge conditions for APPJ, DBD, and other equipment.	25 30
Common plasma generation equipment and technologies	Direct current glow discharge	1. Apply constant high voltage between anode and cathode in low-pressure gas; electrons gain energy in cathode sheath, and ionization is maintained in positive column. 2. Simple device structure, convenient for studying elementary processes and plasma-surface interaction. 3. Pre-ionization or pulse strategy is needed to improve discharge uniformity in atmospheric pressure/small gap scenarios.	1. Gas pressure (mainly low pressure). 2. Spacing between anode and cathode, applied voltage. 3. Pre-ionization intensity (atmospheric pressure scenario).	1. Preparation of small plasma sources. 2. Material pretreatment units. 3. Basic research on plasma-surface interaction.	30
	RF CCP	1. Typical operating frequency 13.56 MHz; energy is injected into plasma via parallel plate electrodes in capacitive coupling mode. 2. Dual-frequency (e.g., 13.56/2 MHz) driving can independently regulate plasma density and ion energy, improving uniformity and process window.	1. RF frequency (mainstream 13.56 MHz). 2. High and low frequency power during dual-frequency driving. 3. Electrode spacing, gas pressure.	1. Low-pressure plasma cleaning, etching/ashing. 2. Material surface activation.	31
	ICP	1. An external induction coil generates an induction electric field in a vacuum chamber, mainly inductively heating electrons. 2. Features high plasma density and low ion bombardment energy. 3. Independent bias can be used to decouple control of plasma density and ion energy.	1. Number of turns and input power of induction coil. 2. Degree of vacuum. 3. Independent bias voltage.	1. High anisotropic etching. 2. High-throughput material surface treatment.	32
	Microwave/ECR plasma	1. Couple energy at microwave frequencies such as 2.45 GHz; achieve efficient electron energy absorption through electron cyclotron resonance (ECR) under matched magnetic field conditions. 2. Easy to obtain high-density, low-pressure plasma.	1. Microwave frequency (mainstream 2.45 GHz). 2. Magnetic field strength and matching degree. 3. Vacuum pressure.	1. Plasma cleaning and activation. 2. Fine thin film deposition and etching.	32
	DBD	1. A dielectric layer is set between at least one electrode and the discharge gap to restrict arc formation and achieve non-equilibrium discharge. 2. Can operate stably at atmospheric pressure with diverse configurations (volume type, surface type).	1. Dielectric layer material (e.g., quartz, ceramic) and thickness. 2. Electrode structure (volume type/surface type). 3. Atmospheric pressure environmental parameters.	1. Atmospheric pressure plasma activation. 2. Continuous material surface treatment. 3. Core technology of atmospheric plasma cleaning equipment.	34
	APPJ/CAPJ	1. Generate plasma plume in the atmosphere through coaxial, needle-ring, or dielectric-coated nozzles to transport short-lived and long-lived active species. 2. Nozzle configuration, working gas, and driving waveform affect ionization wave propagation, chemical field distribution, and species flux.	1. Nozzle configuration (coaxial/needle-ring). 2. Working gas type. 3. Driving waveform parameters (frequency, duty cycle).	1. Material surface modification. 2. Pollutant removal. 3. Preparation and transport of plasma activated gas (PAG).	25 35
	Corona discharge and other atmospheric sources	1. Achieve local discharge under a strong inhomogeneous electric field (e.g., needle-plate electrode). 2. Simple device structure and fast response speed.	1. Electrode structure (needle-plate). 2. Applied voltage intensity (needs to generate strong inhomogeneous electric field).	1. Gas denitrification and purification. 2. Online material surface treatment. 3. One of the active gas generation approaches.	36
	Vacuum plasma cleaner (based on CCP/ICP/ECR)	1. Introduce process gases such as oxygen and argon into the evacuated reaction chamber. 2. Remove organic pollutants and introduce functional groups on the material surface through the combined action of physical sputtering and chemical reaction.	1. Process gas type (oxygen, argon, etc.). 2. Degree of vacuum. 3. Treatment time, discharge power.	Uniform and repeatable cleaning and surface modification of shape and heat-sensitive materials.	33
	Atmospheric plasma cleaning/activation equipment (based on DBD/APPJ)	1. Operate without vacuum and online. 2. Can directly generate and transport PAG to achieve surface activation and decontamination.	1. Working gas type. 2. Discharge power, treatment speed. 3. Spacing between nozzle and surface to be treated.	1. Roll-to-roll continuous process. 2. Treatment of large-area special-shaped parts. 3. Generation and transport unit of PAG.	33 35

APPJ: Atmospheric pressure plasma jet; CAPJ: cold atmospheric plasma jet; CCP: capacitively coupled plasma; DBD: dielectric barrier discharge; ECR: electron cyclotron resonance; He: helium; ICP: inductively coupled plasma; pd: pressure-gap product; PAG: plasma activated gas; RF: radio frequency; *T*_g_: gas temperature; *T*_e_: electron temperature.

## Composition and Speciation of Plasma-Activated Gas

### Generation of primary reactive species

In non-thermal atmospheric-pressure plasmas, energy supplied by an external electric field drives the formation of active species, including electrons, ions, radicals, and excited-state molecules. High-energy electrons undergo inelastic collisions with ground-state molecules or atoms, initiating ionization, dissociation, and excitation processes. Metastable states of rare gases (e.g., He, Ar) further enhance particle density via Penning ionization, sustaining the non-equilibrium discharge.^36^,^37^ The filamentary or streamer structure of the discharge zone governs the transient flux of short-lived species (e.g., O, OH, singlet oxygen, NO) in the gas phase and their transport to interfaces. Concurrently, energy partitioning and waveform modulation within the plasma sheath alter the electron energy distribution, thereby influencing primary reaction pathways and secondary chemical routes.^38^

When the plasma interacts with a liquid phase, gaseous RONS dissolve, become trapped, and undergo cascade reactions at the gas–liquid interface, yielding a tunable mixture of short- and long-lived species. The concentrations of these species are modulated by discharge source type, energy density, treatment duration, ambient humidity, and gas composition.^39^,^40^ In the liquid phase, ·OH radicals may form via the O(¹D) + H_2_O reaction channel or through electron reduction/re-oxidation of dissolved oxygen, while H_2_O_2_ is mainly produced by ·OH recombination or stepwise reactions involving O_2_·^−^. These kinetic processes are strongly influenced by pH, buffer ions, and organic substrates.^41^,^42^ In situ and quasi-in situ analytical techniques, such as transient spectroscopy, scavenger chemistry, and isotope tracing, have validated the existence of a “short-lived to long-lived” cascade reaction network in plasma–liquid systems. These methods have elucidated the timescales and characteristic fluxes of key species, providing an experimental basis for correlating PAG processing parameters with composition and bioactivity.^43^

### Variation in reactive species with feedstock gas

The composition of the inlet gas is the principal determinant of RONS diversity and relative abundance. When rare gases serve as the primary feed, He or Ar, with their high metastable state densities and stable discharge characteristics, efficiently produce a ROS-dominated spectrum (e.g., O, OH, O_3_, H_2_O_2_) upon introduction of trace O_2_ or H_2_O.^44^ Humid air with He favors ·OH generation through energy-transfer channels, whereas Ar with small O_2_ admixtures preferentially yields O atoms and O_3_.^45^,^46^,^47^

When N_2_ or air is introduced, the N_2_(C–B) emission band intensifies, and reactive nitrogen species (RNS) such as NO, NO_2_, and N_2_O become more abundant. In the liquid phase, this leads to accumulation of NO_2_^−^ and NO_3_^−^ and a concomitant decrease in pH. Experiments show that altering the plume atmosphere, for instance, replacing ambient air with pure O_2_ or Ar, markedly suppresses or enhances RNS generation, consequently modulating the inactivation mechanisms and efficacy against bacteria and biofilms.^48^,^49^ In wound treatment based on PAG, RONS are delivered to the wound interface in gaseous diffusion by plasma equipment. Among them, high-valent NO_x_ (such as dinitrogen pentoxide) and gaseous ROS react rapidly with the wound exudate to generate stable liquid-phase active species (such as NO_2_^−^, NO_3_^−^, ONOO^−^). These species can penetrate deeply into the wound tissues and exert antibacterial, anti-inflammatory and healing effects.^8^

**Figure 1:**
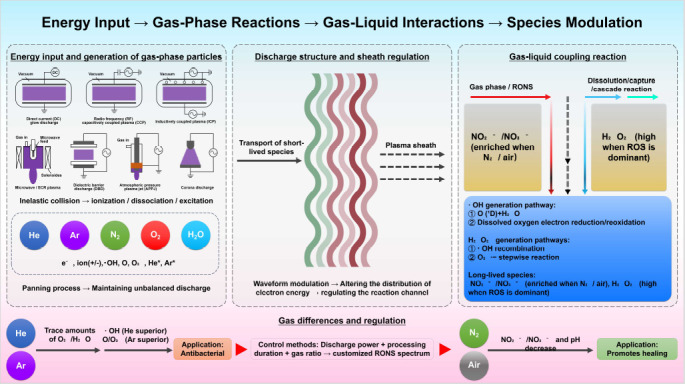
The difference between the generation of active particles and gases in non-thermal atmospheric pressure plasma. It illustrates the multi-stage formation process of PAG, from energy input to gas-liquid coupling reactions, and the regulation of reactive species based on different feedstock gases. Created with Microsoft PowerPoint 2021. Ar: Argon; e^−^: electron; He: helium; H_2_O: water; H_2_O_2_: hydrogen peroxide; OH: hydroxyl radical; N_2_: nitrogen; NO_2_^−^/NO_3_^−^: nitrite anion/nitrate anion; O_2_: oxygen; O_3_: ozone; ROS: reactive oxygen species; RONS: reactive oxygen and nitrogen species.

In PAG applications, the combination of energy density and gas composition enables tailored chemistry, ranging from “ROS-dominant” to “ROS–RNS synergistic” regimes. By adjusting discharge power, treatment time, and gas mixing ratios, one can directionally tune the relative yields of H_2_O_2_ *versus* NO_2_^−^/NO_3_^−^ under controlled pH and redox potential, thereby customizing the RONS profile for specific applications, such as antimicrobial treatment or wound healing.^50^ The difference between the generation of active particles and gases in non-thermal atmospheric pressure plasma is shown in **Figure 1**.

## Biological Effects of Plasma-Activated Gas on Wounds

### Enhancement of wound healing

As a key manifestation of low-temperature plasma technology, PAG exerts multifaceted regulatory effects on the wound microenvironment, cellular repair pathways, and tissue metabolism, demonstrating significant beneficial roles across the critical phases of hemostasis, proliferation, and remodeling. Its efficacy exhibits wound-type adaptability and considerable clinical translational potential. Although the optimal dose of PAG (for example, 3 minutes of treatment per day) can promote healing, excessively high concentrations of PAG or prolonged treatment (≥ 10 minutes per day) may induce cytotoxicity by disrupting the cellular reduction-oxidation balance, leading to delayed wound healing and intensified tissue inflammation.^7^,^8^,^9^

During the hemostatic phase, PAG accelerates coagulation by activating the clotting cascade and enhancing platelet function. Studies indicate that RNS in PAG, particularly nitric oxide (NO), promotes platelet adhesion and aggregation. Specifically, NO binding to soluble guanylyl cyclase on platelets activates the cyclic guanosine monophosphate–protein kinase G signaling axis, inducing activation of platelet membrane glycoproteins such as GPIIb/IIIa and strengthening fibrinogen binding.^51^ In a rat tail transection model, PAG treatment reduced bleeding time by 40–50% compared with saline controls and significantly improved clot stability, correlating with shortened prothrombin time and activated partial thromboplastin time.^22^

Throughout wound repair, PAG not only primes subsequent inflammatory cell recruitment^52^ but also plays a central role in the proliferative phase by driving cell proliferation, migration, and angiogenesis. In keratinocytes (HaCaT cells), ROS in PAG (e.g., H_2_O_2_ and OH^−^) activate the epidermal growth factor receptor (EGFR)–extracellular regulated protein kinases 1/2 (ERK1/2) pathway, accelerating cell cycle progression from G_0_/G_1_ to S phase and enhancing epithelial coverage.^53^ Scratch assays revealed that direct 1-minute PAG treatment increased HaCaT migration by 60–70% and upregulated proliferation markers Ki-67 antigen and proliferating cell nuclear antigen by 2–3 fold.^54^ In fibroblasts, PAG-derived NO induces transforming growth factor-β1 (TGF-β1) secretion, which activates Smad2/3 signaling to stimulate collagen and extracellular matrix (ECM) synthesis and potentiate cell migration.^55^ In a diabetic mouse full-thickness wound model, PAG elevated fibroblast migration rates by 50–60% and increased granulation tissue thickness by 40–50%.^22^ Regarding angiogenesis, PAG promotes neovascularization by modulating VEGF and VEGF receptor expression. PAG-supplied NO_3_^−^ is converted intracellularly to NO, activating the endothelial nitric oxide synthase pathway and enhancing VEGF secretion and vascular endothelial growth factor receptor phosphorylation.^56^ In the chick chorioallantoic membrane model, PAG elevated vessel density by 70–80%, increased branching, and widened vessel diameter.^53^ A rabbit ear ischemic ulcer model further confirmed that PAG improved wound vascularization by over 60%, restoring ischemic zone perfusion to 80% of normal within 7 days.^55^

In the remodeling phase, PAG optimizes ECM degradation and restructuring by rebalancing matrix metalloproteinases (MMPs) and their tissue inhibitors (TIMPs), thereby supporting functional tissue recovery. Under physiological healing, MMPs (e.g., MMP-1 and MMP-9) remove damaged ECM, while TIMPs (e.g., TIMP-1) prevent excessive proteolysis. PAG treatment significantly downregulated MMP-9 expression by 40–50% in chronic wounds while upregulating TIMP-1 by 2–3 fold, thereby limiting ECM degradation and promoting collagen crosslinking and tissue strength.^53^ In a rat cutaneous scar model, PAG reduced scar thickness by 30–40% and normalized the collagen I/III ratio (PAG: 3.5–4.0 *vs*. control: 2.0–2.5), indicating its potential to mitigate fibrotic outcomes.^55^

Moreover, PAG demonstrates broad therapeutic activity across wound types. In acute wounds (e.g., surgical incisions), it shortened healing time by 25–35% and reduced postoperative infection risk.^54^ In chronic wounds such as diabetic foot ulcers, PAG improved the wound milieu, reduced bacterial load, and enhanced cellular activity, increasing healing rates by 50–60%.^22^ In burn wounds, it attenuated inflammation, promoted re-epithelialization, and suppressed scar hyperplasia.^53^ A clinical pilot study of 22 patients with chronic wounds confirmed that PAG treatment reduced healing time by 30–40% and significantly lowered visual analogue scale score.^56^

### Anti-infective performance

Against Gram-positive bacteria, such as *S. aureus* and methicillin-resistant *S. aureus* (MRSA), key clinical challenges, PAG exhibits potent bactericidal effects. *In vitro*, a 9-minute PAG treatment reduced viable counts by > 8 log_10_ colony-forming unit, with comparable efficacy against MRSA and susceptible strains.^51^ This action arises from synergistic effects of multiple reactive species: OH^−^ directly oxidizes phospholipid bilayers, increasing membrane permeability; O_2_^−^ induces lipid peroxidation, generating malondialdehyde and disrupting membrane integrity; and O_3_ penetrates the cell membrane, oxidizing intracellular protein thiols and nucleic acid bases, thereby inhibiting bacterial metabolism and DNA replication.^52^ In a murine dorsal infection model, PAG reduced wound bacterial load by 6–7 log_10_ versus saline controls and significantly suppressed pro-inflammatory cytokines, tumor necrosis factor α and interleukin 6 by 50–60%.^22^

PAG is equally effective against Gram-negative bacteria, which are protected by an outer membrane rich in lipopolysaccharide. *In vitro*, 9-minute PAG exposure reduced *Pseudomonas aeruginosa* (*P. aeruginosa*) and *Escherichia coli* (*E. coli*) counts by 8 log_10_ and 7 log_10_ colony-forming unit, respectively.^51^ Mechanistically, NO_2_^−^ in PAG binds to lipopolysaccharide, compromising outer membrane integrity, while the acidic PAG environment (pH 2.4–3.5) enhances membrane permeability, facilitating penetration of reactive species.^52^ In a rat *P. aeruginosa* infection model, PAG shortened healing time by 40–50% and reduced bacterial burden in wound exudates to below detectable levels within 7 days.^53^

A central advantage of PAG lies in its ability to disrupt biofilms, structured bacterial communities that resist conventional antibiotics. PAG attacks biofilms via dual pathways: direct oxidation of the extracellular polymeric substance matrix, reducing biofilm adhesion, and eradication of embedded bacteria, inhibiting proliferation and dissemination.^51^ In a *S. aureus* biofilm model, PAG treatment for 9 minutes reduced biomass by 80–90% and decreased viable biofilm bacteria by 8 log_10_ colony-forming unit.^52^ A clinical case report described a diabetic foot ulcer with MRSA biofilm infection that had failed to heal for 6 months; after 6 weeks of PAG therapy, the biofilm was eradicated, bacterial cultures turned negative, and complete wound closure was achieved by week 12.^55^

**Figure 2:**
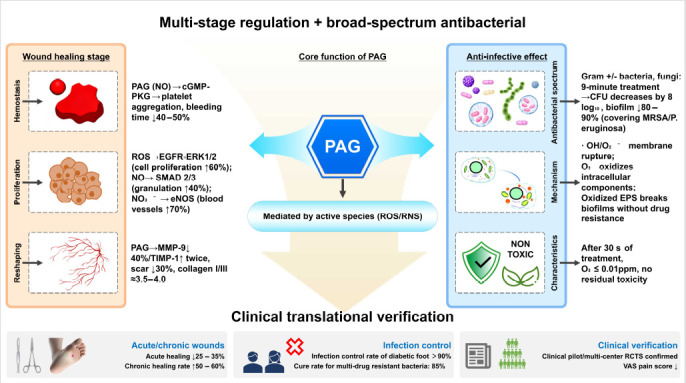
Multi-stage regulation, broad-spectrum antibacterial properties and clinical translation of PAG. It illustrates PAG’s dual functions in wound healing by regulating hemostasis, proliferation, and reshaping stages and in anti-infection through broad-spectrum antibacterial activity and safety as well as its clinical translational verification. Created with Microsoft PowerPoint 2021. CFU: Colony-forming unit; cGMP: cyclic guanosine monophosphate; EGFR: epidermal growth factor receptor; eNOS: endothelial nitric oxide synthase; EPS: extracellular polymeric substance; ERK1/2: extracellular regulated protein kinases 1/2; MRSA: methicillin-resistant *Staphylococcus aureus*; MMP-9: matrix metalloproteinase-9; *P. aeruginosa*: *Pseudomonas aeruginosa*; PAG: plasma-activated gas; PKG: protein kinase G; RCTs: randomized controlled trials; RNS: reactive nitrogen species; ROS: reactive oxygen species; SMAD: small mother against decapentaplegic; TIMP-1: tissue inhibitor of metalloproteinases-1; VAS: visual analog scale.

The anti-infective efficacy of PAG has been validated in multiple preclinical and clinical studies. A randomized controlled trial of 45 diabetic foot ulcer patients reported a 7–8 log_10_ reduction in wound bacterial load with PAG, achieving > 90% infection control rates compared to 50% in the control group.^51^ A separate multicenter randomized controlled trial showed that PAG combined with standard care resulted in an 85% infection resolution rate in patients with multidrug-resistant wound infections, significantly outperforming the 40% rate with standard care alone.^55^ Notably, PAG exerts rapid and sustained antimicrobial action without residual toxicity: its ozone concentration decays to < 0.01 ppm within 30 seconds post-treatment, underscoring its clinical safety profile.^51^ The multi-stage regulation, broad-spectrum antibacterial properties, and clinical translation verification of PAG are shown in **Figure 2**.

## Mechanistic Insights into Plasma-Activated Gas

### Cellular-level mechanisms

PAG orchestrates wound healing by precisely modulating the biological behavior of distinct cell types within the wound microenvironment through specific signaling pathways. Its effects exhibit clear cell-type specificity and are critically dependent on reactive species concentration and exposure duration.

#### Promotion of cell proliferation

PAG enhances the proliferation of three major wound-associated cell types through distinct mechanisms: (1) Keratinocytes: H_2_O_2_ in PAG activates the EGFR–ERK1/2 pathway, upregulating CyclinD1 and Cdk2 expression and accelerating cell cycle progression from G_0_/G_1_ to S phase.^53^ In HaCaT cells, PAG containing 1.74 μM H_2_O_2_ increased proliferation by 2–3 fold, an effect substantially inhibited (60–70% reduction) by the EGFR inhibitor Gefitinib.^54^ (2) Fibroblasts: PAG-derived NO stimulates guanylyl cyclase, elevating cyclic guanosine monophosphate levels and activating protein kinase G, which upregulates proliferation-associated genes, such as c-myc.^56^ PAG enhanced fibroblast proliferation by 1.5–2 fold, reversible by nitric oxide synthase inhibitor N^5^-nitro-L-arginine methyl ester (50–60% decrease).^55^ (3) Endothelial cells: Intracellular conversion of PAG-supplied NO_3_^−^ to NO enhances VEGF receptor phosphorylation, activating the phospholipase Cγ/protein kinase C axis to promote endothelial proliferation.^22^

#### Acceleration of cell migration

PAG facilitates cellular migration by remodeling the cytoskeleton and regulating adhesion molecules: (1) Keratinocytes: PAG activates Rac1 and Cdc42, inducing filopodia and lamellipodia formation, enhancing actin polymerization, and downregulating intercellular adhesion molecules such as E-cadherin.^53^,^54^ Scratch assays demonstrated 60–70% faster migration and more organized actin fibers.^52^ (2) Fibroblasts: PAG-induced TGF-β1 secretion activates Smad2/3 signaling, increasing MMP-2 expression to degrade collagen fibers and create migration paths through the ECM.^55^ (3) Endothelial cells: OH^−^ in PAG activates integrin β1, strengthening ECM adhesion and promoting cytoskeletal reorganization to direct endothelial migration toward ischemic areas.^22^

#### Regulation of cell differentiation

PAG drives functional differentiation through key signaling pathways. In keratinocytes, PAG-activated Notch signaling promotes epidermal differentiation, elevating keratin 1 and keratin 10 expression by 2–3 fold and enhancing barrier function.^54^,^56^ H_2_O_2_ in PAG activates the TGF-β1/Smad3 pathway, inducing myofibroblast differentiation marked by a 3–4 fold increase in alpha-smooth muscle actin expression, facilitating wound contraction.^52^,^53^ PAG-derived NO stimulates the endothelial nitric oxide synthase pathway, promoting VEGF secretion and guiding endothelial differentiation into mature vascular structures.^22^

### Molecular-level mechanisms

The rich repertoire of RONS in PAG mediates both bactericidal and pro-healing effects through targeted interactions with essential biomolecules, including DNA, proteins, and lipids, in bacteria and wound cells. These interactions involve oxidative modifications, structural disruption, and functional regulation, demonstrating multi-target synergistic characteristics.

#### Bactericidal activity

PAG achieves efficient microbial eradication via coordinated attacks on core bacterial biomolecules. Reactive species in PAG directly damage bacterial genetic material. OH^−^, for instance, oxidizes DNA bases such as guanine to form 8-hydroxy-2′-deoxyguanosine, leading to strand breaks and base mispairing that ultimately inhibit DNA replication and transcription.^52^ *In vitro* studies confirmed that PAG treatment of *S. aureus* increased DNA fragmentation by 3–4 fold and elevated 8-hydroxy-2′-deoxyguanosine levels by 2–3 fold.^51^ Additionally, NO_2_^−^ from PAG reacts with amino groups in DNA, generating N-nitroso compounds that distort DNA structure and impede DNA polymerase binding.^22^ For Gram-negative *E. coli* (a common pathogen causing abdominal and wound infections), the accumulation of 8-OHdG induced by PAG can impair the fidelity of DNA replication. For fungal pathogens, such as *Candida albicans* (which often causes superficial wounds and mucosal infections), RONS derived from PAG disrupt the integrity of mitochondrial DNA, inhibit energy metabolism, and ultimately suppress fungal proliferation.^57^ Bacterial proteins represent another key target. O_2_^−^ oxidizes critical thiol groups (–SH) to disulfide bonds (–S–S–), causing protein denaturation and loss of function.^52^ PAG treatment reduced DNA polymerase activity in S. aureus by 60–70%, while H_2_O_2_ oxidized tyrosine residues to 3-nitrotyrosine, altering protein structure and activity.^51^ In MRSA, PAG oxidizes the thiol group in penicillin-binding protein 2a, a core protein mediating antibiotic resistance, reducing its activity and restoring bacterial sensitivity to β-lactam antibiotics.^57^ OH^−^ and O_2_^−^ induce lipid peroxidation in bacterial membranes, generating malondialdehyde and other harmful products that compromise membrane integrity.^52^ In *P. aeruginosa*, PAG elevated malondialdehyde content by 2–3 fold and increased membrane permeability by 40–50%, resulting in leakage of K^+^ ions and nucleic acids, ultimately causing cell death.^51^ For *Acinetobacter baumannii* (a Gram-negative pathogen associated with nosocomial wound infections), PAG treatment increases membrane permeability and raises malondialdehyde levels, leading to intracellular protein leakage and cell lysis.^56^,^57^

#### Wound healing

While neutralizing pathogens, PAG simultaneously promotes tissue repair by precisely modulating functional biomolecules in host cells. PAG modifies key signaling proteins to activate pro-healing pathways. H_2_O_2_ oxidizes tyrosine residues in the EGFR, activating the EGFR signaling axis and promoting cell proliferation and migration.^53^ *In vitro*, PAG elevated EGFR phosphorylation by 2–3 fold and downstream ERK1/2 phosphorylation by 3–4 fold.^54^ Concurrently, NO from PAG activates soluble guanylyl cyclase, increasing cyclic guanosine monophosphate production and activating protein kinase G, which upregulates VEGF expression to stimulate angiogenesis.^56^ In fibroblasts, this pathway increased VEGF mRNA and protein levels by 3–4 fold and 2–3 fold, respectively.^55^ PAG modulates MMP activity to degrade damaged collagen and fibronectin, clearing paths for cell migration and tissue reconstruction.^53^

Beyond the above, PAG exerts therapeutic effects through two key regulatory axes and realizes pathway crosstalk to ensure orderly wound healing. On one hand, ROS from PAG dissociates nuclear factor erythroid 2-related factor 2 (Nrf2) from Kelch-like ECH-associated protein 1 (Keap1), enabling nuclear translocation and antioxidant response element binding to upregulate antioxidant enzymes (e.g., heme oxygenase-1, NAD(P)H quinone dehydrogenase 1). In diabetic wounds, PAG elevated heme oxygenase-1 by 3–4 fold and reduced oxidative stress by 50–60%.^54^,^55^ On the other hand, RNS in PAG suppresses nuclear factor κB activation, reducing expression of tumor necrosis factor α and interleukin 6. In a rat inflammation model, PAG decreased nuclear factor κB activity by 40–50% and markedly reduced neutrophil and macrophage infiltration.^19^,^53^,^54^

Notably, there may be certain cross-talk among the various signaling pathways (EGFR, TGF-β, etc.) activated by the PAG mentioned above. For instance, ROS induced by PAG activates the Nrf2/antioxidant response element antioxidant pathway to alleviate oxidative stress damage, thereby maintaining keratinocyte proliferation mediated by the EGFR/ERK1/2 pathway. Meanwhile, the TGF-β1/Smad3 pathway, in promoting the differentiation of fibroblasts into myofibroblasts and the synthesis of collagen, works in synergy with the inhibitory effect of the nuclear factor κB pathway to achieve a dynamic balance between inflammatory response and tissue repair.^8^ Despite this, the coordinated regulation among pathways can enable PAG to simultaneously promote the processes of cell proliferation, migration, and differentiation at the tissue level, thereby effectively ensuring the orderly connection of each stage from hemostasis to remodeling of the wound.

## Limitations and Challenges

### Technical challenges

PAG technology faces several core technical bottlenecks. The primary challenge lies in controlling and tracing active species. Variations in plasma source type, power waveform, and gas composition significantly alter electron energy distributions and primary/secondary reaction pathways, leading to substantial disparities in the resulting PAG profiles, including reactive species composition (e.g., O, OH, O_3_, and NO_x_), near-surface pH, and oxidation-reduction potential. The absence of standardized dosimetry and reliable online monitoring methods severely compromises reproducibility and cross-laboratory comparability.^57^

At the device engineering level, developing stable, low-power, portable miniaturized plasma sources with closed-loop control remains a focal point. While continuous gas feeding and online activation (e.g., via jet recirculation or sealed chambers) can improve PAG consistency, these approaches impose stricter demands on reactor mass transfer, thermal management, and long-term operational reliability.^56^

**Figure 3:**
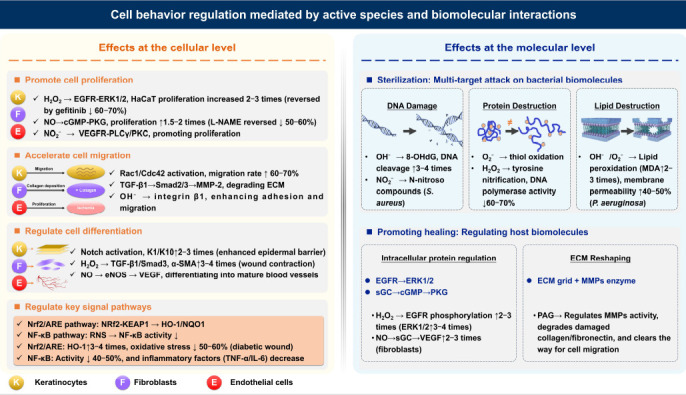
The mechanism of PAG at the cellular and molecular levels. It illustrates the regulatory effects of PAG on the behavior of keratinocytes, fibroblasts, and endothelial cells at the cellular level, including promoting proliferation, migration, and differentiation. Meanwhile, it shows the multi-target antibacterial mechanism of PAG and its role in regulating host biomolecules to promote healing at the molecular level. Created with Microsoft PowerPoint 2021. Cdc42: Cell division control protein 42; ECM: extracellular matrix; EGFR: epidermal growth factor receptor; eNOS: endothelial nitric oxide synthase; ERK1/2: extracellular regulated protein kinases 1/2; GMP: guanosine monophosphate; HO-1: heme oxygenase-1; H_2_O_2_: hydrogen peroxide; IL-6: interleukin-6; iNOS: inducible nitric oxide synthase; L-NAME: Nω-nitro-L-arginine methyl ester; MDA: malondialdehyde; MMP: matrix metalloproteinase; NF-κB: nuclear factor kappa-B; Nfr2/ARE: nuclear factor erythroid 2-related factor 2/antioxidant response element; NQO1: NAD(P)H quinone dehydrogenase 1; NO: nitric oxide; NO_2_^−^: nitrite anion; NO_3_^−^: nitrate anion; O_2_^−^: superoxide anion; 8-OHdG: 8-hydroxy-2’-deoxyguanosine; OH: hydroxyl radical; PAG: plasma-activated gas; *P. aeruginosa*: *Pseudomonas aeruginosa*; PLCγ: phospholipase C gamma; PKG: protein kinase G; Rac1: Ras-related C3 botulinum toxin substrate 1; sGC: soluble guanylyl cyclase; *S. aureus*: *Staphylococcus aureus*; Smad: small mother against decapentaplegic; TGF-β1: transforming growth factor-beta 1; TNF-α: tumor necrosis factor-alpha; VEGFR: vascular endothelial growth factor receptor; VEGF: vascular endothelial growth factor; α-SMA: alpha-smooth muscle actin.

Furthermore, fundamental mechanistic understanding remains incomplete. The complex chemistry at gas–liquid–bio interfaces and the insufficient characterization of short-lived reactive species via in situ techniques hinder the establishment of precise correlations between process parameters, chemical composition, and biological effects, thus limiting technological optimization and application. The mechanism of action of PAG at the cellular and molecular levels is shown in **Figure 3**.

### Clinical translation progress, challenges, and prospective strategies of plasma-activated gas

In a Phase 1 clinical trial approved by the U.S. Food and Drug Administration conducted at the Rush University Medical Center, a pen-like atmospheric pressure plasma device (capable of generating PAG) was used as an adjunctive treatment for recurrent myxofibrosarcoma. The room-temperature plasma gas was delivered to the residual tumor site to induce cancer cell death while avoiding damage to normal tissues, thereby addressing the risks of adjuvant treatment for elderly patients. Additionally, there are 13 trials registered at Clinicaltrials.gov regarding plasma-activated media, including PAG, focusing on wound healing and skin diseases, laying the foundation for standardization and transformation.

For U.S. Food and Drug Administration-approved instruments, the Renuvion helium plasma system of Axmed Medical Company generates helium PAG through radiofrequency energy for soft tissue cutting/coagulation; its dual-mode energy delivery technology can achieve safe tissue contraction, and its non-label skin regeneration effect is comparable to that of laser skin resurfacing.^58^ In 2024, the UNIPURE C3F8 ophthalmic gas and its delivery system adopted a targeted gas delivery method similar to PAG, and the retinal reattachment rate reached 81.9% at 3 months, verifying the safety of gas delivery.^59^ Although there are no products specifically for PAG wound care equipment that have received approval from the US Food and Drug Administration, these precedents provide strong support for the further advancement of PAG-related regulatory work.

Despite such progress, the clinical adoption of PAG confronts multiple challenges. (1) Safety evaluation: Systematic, long-term longitudinal follow-up studies and tissue-specific safety assessments are still needed to evaluate risks of excessive oxidative stress or genotoxicity from prolonged or repeated PAG exposure. (2) Treatment standardization: The entire therapeutic workflow lacks unified quality control standards, covering PAG preparation parameters, storage and usage timelines, and clinical protocols (e.g., irrigation or dressing frequency and duration), undermining the strength and comparability of clinical evidence. (3) Implementation realities: Practical factors such as insurance reimbursement policies, training of healthcare staff, and adaptation of existing clinical workflows must be addressed before PAG can be integrated into routine care. In terms of economic feasibility, the current initial investment cost of equipment for PAG-related therapies is higher than that of traditional wound care and antibacterial treatment methods. Moreover, long-term cost-benefit data (such as shortening hospital stays and reducing recurrence rates) are still relatively scarce.

To promote clinical transformation, targeted technical and implementation suggestions include: (1) Developing intelligent PAG devices with real-time monitoring and personalized dose control; (2) Establishing a multi-center collaborative platform to unify data standards and guide the formulation of guidelines; (3) Promoting portable low-cost devices and hierarchical clinical training to address manufacturing scalability and reduce per-unit costs; (4) Conducting a health economic assessment to support reimbursement policies and clarify regulatory approaches to strengthen economic feasibility arguments. To advance clinical translation, recent reviews and translational studies recommend prioritizing prospective randomized controlled trials and stratified studies, establishing feasible dose–response and risk–benefit frameworks, and simultaneously clarifying regulatory pathways and developing clinical consensus guidelines.^60^

## Conclusion and Perspectives

As a prominent application of low-temperature plasma technology, PAG demonstrates considerable advantages in wound healing and infection control. In tissue repair, PAG exerts coordinated therapeutic effects across multiple healing phases: it activates platelets and coagulation factors to reduce bleeding time by 40–50% during haemostasis^51^; enhances keratinocyte and fibroblast proliferation and migration, increasing granulation tissue thickness by 40–50%, while boosting wound vascular density by 70–80% via NO-mediated endothelial nitric oxide synthase signaling during proliferation^51^,^52^; and rebalances MMPs and TIMPs to reduce scar thickness by 30–40% during remodeling.^54^

In antimicrobial applications, PAG exhibits broad-spectrum efficacy against both Gram-positive (e.g., *S. aureus*, MRSA) and Gram-negative bacteria (e.g., *P. aeruginosa, E. coli*), achieving > 8 log_10_ reduction in bacterial load within 9 minutes of treatment and disrupting biofilm architecture, reducing biomass by 80–90%.^50^,^51^

Mechanistic studies reveal that PAG’s bioeffects stem from synergistic interactions between RONS. At the cellular level, PAG activates key signaling pathways, including EGFR–ERK1/2 and Nrf2/antioxidant response element, to precisely regulate proliferation, migration, and differentiation.^53^,^54^ Molecular analyses show that PAG inactivates microbes through oxidative damage to DNA (e.g., 8-hydroxy-2′-deoxyguanosine formation), proteins (thiol and tyrosine oxidation), and lipids (peroxidation), while simultaneously promoting tissue repair by modulating expression of cytokines such as VEGF and TGF-β1.^22^,^50^,^51^ Preliminary studies in animal models (e.g., diabetic db/db mice, rat ischemic ulcers) and small-scale clinical trials have validated PAG’s safety and efficacy, though further development faces both technical and clinical challenges.^54^,^55^

Future advances in PAG research and application require progress on multiple fronts. First, establishing multidimensional detection and quantification systems is essential. Integrating real-time in situ techniques (e.g., Raman spectroscopy, time-resolved fluorescence imaging) with mathematical modelling will help quantify dynamic relationships between discharge parameters (voltage, gas flow) and reactive species concentrations/ratios, enabling identification of optimal reactive species “fingerprints” for specific wound types (acute/chronic, infected/non-infected).54 Machine learning could assist in identifying ideal species profiles, for instance, an OH^−^/NO_2_^−^ ratio of 2:1 with H_2_O_2_ concentrations of 1.5–2 μM for diabetic foot ulcers.^51^ Deeper investigation into PAG’s crosstalk with cellular signaling pathways, such as ROS-mediated epigenetic regulation of wound repair genes via histone or DNA methylation, may reveal novel targets for precision therapy.^53^

Second, developing integrated, intelligent PAG devices represents a critical engineering goal. Microelectromechanical systems could miniaturize discharge units, and portable gas cartridges (e.g., micro-argon tanks) may help reduce device weight below 5 kg, facilitating bedside use.^50^,^54^ Closed-loop control systems that automatically adjust discharge parameters based on real-time monitoring of wound pH and reactive species concentrations would enhance treatment consistency and reproducibility,^53^ for example, reducing NO_2_^−^ generation when pH falls below 3.0 to prevent tissue damage.^51^ Multimodal PAG systems incorporating complementary functions (e.g., combining antimicrobial and pro-healing actions with low-frequency pulsed electric fields to alleviate pain via nerve modulation) could improve patient comfort and acceptance.^55^

Clinically, key priorities include establishing standardized PAG treatment guidelines specifying optimized parameters for different wounds (e.g., 1 min/session daily for acute incisions; 5 min/session every 3 days for diabetic ulcers) and conducting large-scale, multicenter randomized controlled trials (≥ 500 patients) to verify long-term safety and efficacy.^54^ Expanding PAG’s indications to complex wounds such as burns (preventing scar hyperplasia) and radiation ulcers (improving microcirculation) is promising. Investigating synergies between PAG and advanced therapies, for instance, PAG pretreatment enhancing mesenchymal stem cell migration to improve healing rates by 15–20%,^52^,^54^ may open new combinatorial strategies. Finally, facilitating insurance reimbursement through rigorous health-economic analyses (e.g., demonstrating a 30% reduction in amputation risk and 25% cost savings for diabetic foot ulcers) will be crucial for broadening clinical access to this innovative technology.^51^

## Data Availability

*Not applicable.*
